# Tefillin use induces preconditioning associated changes in heart rate variability

**DOI:** 10.1371/journal.pone.0280216

**Published:** 2023-01-18

**Authors:** Sheryl E. Koch, Elyse Martin, Amitesh Verma, Stacey Adjei, Jack Rubinstein

**Affiliations:** Department of Internal Medicine, Division of Cardiovascular Health & Disease, University of Cincinnati College of Medicine, Cincinnati, Ohio, United States of America; Nippon Medical School Graduate School of Medicine, JAPAN

## Abstract

Short bouts of occlusion of blood flow can induce a preconditioning response that reduces subsequent damage from longer periods of ischemia. It has been shown that ischemic preconditioning (IPC) can be elicited remotely (RIPC) through limitation of blood flow and as recently described via only pain sensation. Non-obstructive banding (NOB) through the donning of tefillin (a box with sacred texts attached to a leather strap that is traditionally bound to the non-dominant arm of Jewish adults during morning prayers) has been shown to elicit an RIPC response at least partially through pain sensation. This study evaluated the effects of NOB on heart rate variability (HRV) dependent factors that are known to be affected by various RIPC stimuli. We recruited 30 healthy subjects and subjected them to NOB versus control and found various HRV markers associated with RIPC to be changed in the NOB group. This finding provides further evidence that tefillin, likely through NOB induced RIPC changes, may still be a viable clinical pathway to prevent and decrease the morbidity associated with ischemic events.

## Introduction

It is well established that short bouts of occlusion of blood flow can induce a preconditioning response that reduces subsequent damage from longer periods of ischemia to the same organ [1–5]. When this effect is generated distant from the subsequently injured tissue, it is described as remote ischemic preconditioning (RIPC) [6–8] and has been shown in animal models to result in dramatically decreased infarct size when applied prior to an ischemic myocardial event [9–13]. Unfortunately, despite decades of research and dozens of randomized patient trials, this effect has not yet been translated into positive human studies [14–19].

Our laboratory and others have investigated the RIPC effect in different animal [20, 21] and human conditions [19, 22] and have found that ischemia itself is not necessary to induce RIPC as the effect appears to be mediated by pain sensation and at least partially through transient potential receptor vanilloid (TRPV) channel stimulation [20, 21]. Further, we have documented that the effect can be elicited by non-obstructive banding (NOB) of an extremity in healthy male individuals through the use of tefillin, which is a box with sacred texts attached to a leather strap that is traditionally bound to the non-dominant arm of Jewish adults during morning prayers [23]. The strap is usually worn for approximately 30 minutes in a manner that is tight enough to result in indentations in a helical manner on the skin of the arm and forearm but not tight enough to completely obstruct blood flow to the extremity. The preconditioning effect was noted to improve vascular and inflammatory markers that are strongly associated with RIPC phenotype and was subsequently shown in an animal model to decrease infarct size through pain mediated pathways even when the blood flow to the extremity was not completely occluded [20]. The sum of these effects was put into the context of several population based studies that earlier had identified orthodox Jewish males as having lower risk of coronary heart disease death (even after controlling for key variables), by proposing that an important factor was their near daily use of an NOB inducing RIPC phenotype [24].

This study focused on further elucidating the pathways involved in the NOB induced RIPC response by including both males and females and by focusing on the RIPC effects on heart rate variability (HRV) derived values. Prior studies with healthy subjects as well as patients with cardiovascular diseases have shown that various RIPC protocols are capable of eliciting changes in HRV derived parameters [25–32]. HRV is determined by measuring the beat-to-beat (RR) intervals and reflects cardiac autonomic modulation. HRV indices include time domain variables, such as standard deviation of all normal RR intervals (SDNN), the percentage of successive RR intervals that differ by more than 50 ms (pNN50), the root mean square of successive RR interval differences (RMSSD) and the HRV triangular index which is the integral of the density of the RR interval histogram divided by its height. Other HRV derived parameters include the frequency dependent variables such as low and high frequency power (LF and HF) which are quantified by changing the observed variation into Hertz and lastly the non-linear measurements SD1 and SD2 which are obtained from a Poincare plot of the SDNN.

HRV has been studied as a prognostic marker in cardiac events, ischemic heart disease and heart failure [33, 34]. The Framingham Heart Study found that for at least one subgroup of patients there was a strong correlation between time domain markers of HRV such as SDNN and (less so pNN50) as well as frequency domain markers such as LF and HF [33] and cardiac events [33]. Concerning outcomes after an ischemic event, most studies have found correlation between lower SDNN and poor outcomes as well as between other linear, fractal, and spectral domain measures [34–37]. Similar, though not as consistent, findings have been reported in patients with heart failure [34].

The time domain variables indicate the amount of HRV. Lower time domain values reflect an autonomic dysfunction, while higher values could mean a more healthy autonomic function [34]. SDNN is the “gold standard” of HRV time domain measurements and can be used to predict morbidity and mortality in healthy and diseased states, while pNN50 is closely correlated with parasympathetic nervous system (PNS) activity [33]. Frequency domain measurements quantify the amount of variance in HR. Low values are associated with a lack of autonomic modulation, though higher values cannot be assumed to reflect a superior HRV [34]. LF is associated with PNS and sympathetic nervous system (SNS) activity while HF power is associated with PNS activity. The non-linear measurements reflect baroreceptor activity, including SD1 which is associated with short term HRV and SD2 which is associated with long term HRV and tends to correlate with LF [38].

We hypothesized that the RIPC phenotype induced by NOB tefillin use extended beyond the inflammatory and vascular pathways that we had previously described and is directly associated and would be immediately measured with improved HRV variables. We randomly assigned 30 healthy subjects to either traditionally bound tefillin or a nearly identical control group where the tefillin were placed, but loosely tied. Subjects were not informed or made aware in any way regarding the expected effects. In all cases, the tefillin used was identical to those worn traditionally in the Jewish religion but without the sacred text that is held within the box.

## Methods

### Participants

We recruited 30 healthy subjects (males n = 14; females n = 16) 18 to 40 years of age from the greater Cincinnati area; specifically, they were recruited from around the Cincinnati area. The University of Cincinnati Institutional Review Board (no. 2019–1354) approved all study procedures and was then registered on the UCHealth Research Clinical Study website under the title “Effect of tefillin (phylacteries) on changes in heart rate in healthy people”.

### Study design

**Fig 1:**
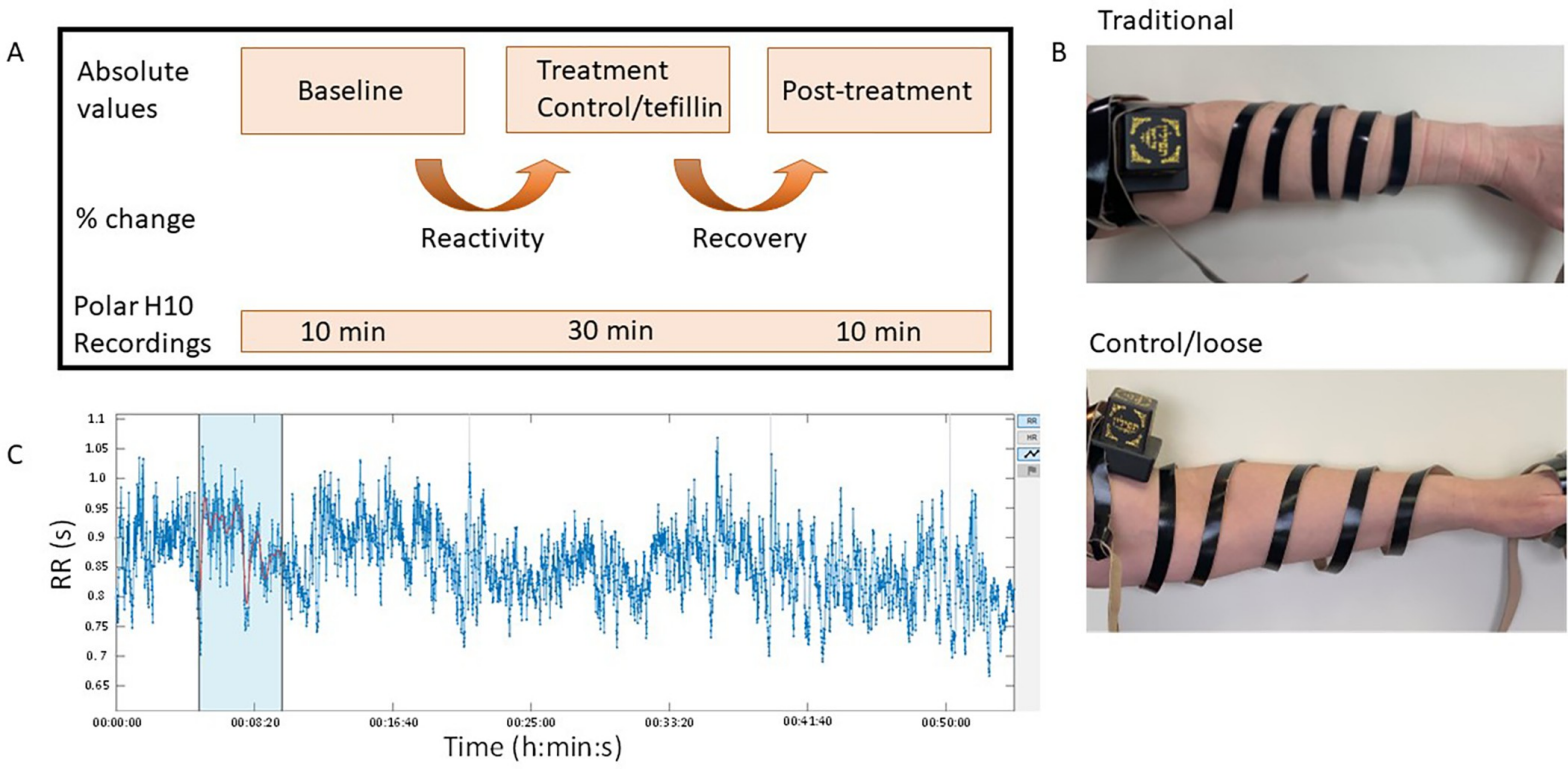
Experimental design and software analysis. **A.** Diagram of the experimental design with the time points (baseline, treatment and post-treatment) as well as the change from baseline to treatment (reactivity) and from treatment to post-treatment (recovery) for control and tefillin groups. B. Depiction of the traditional and control tefillin wrapping. C. Representative graph of the processed HRV data in the Kubios software before analysis. Blue shaded area demonstrates the 5-minute window used for analysis.

All subjects gave written informed consent and completed a questionnaire about their health. Inclusion criteria was defined as healthy individuals between the ages of 18 and 40 years old. Major exclusion criteria were history of tobacco use, a reported history of congenital heart disease, prior heart transplantation, pacemaker, severe anemia, any disease or illness that requires daily medication, active participation in another clinical trial, pregnancy, taken caffeine in last 12 hours, or worn tefillin at least once in the last six months. Subjects wore a Polar H10 (Polar Oy, Kempele, Finland) heart rate sensor around their chest to record the heart rate variability. The Polar H10 monitor has been compared to ECG and Holter monitor readings and was shown to detect over 95% of all RR intervals with a 2ms resolution (manufacturer’s product pamphlet). The data was collected through the Elite HRV (Asheville, NC) application. The subjects sat quietly in a room where they sat as still as possible. There were three phases for the recordings. The first was a 10-minute baseline phase, then a 30-minute treatment phase, and lastly a 10-minute post-treatment phase as diagrammed in **Fig 1A.** Between each phase, there was a designated 2-minute period to put on and remove the tefillin. The change between baseline and treatment is referred to as the reactivity phase and the change between the treatment and post-treatment as the recovery phase. Subjects were randomized into one of two groups. The first group wore the tefillin in a traditional manner that did not fully occlude the blood flow, but tight enough to induce small indentations on the skin surface in a manner as similar as possible to traditional Jewish custom while the second group wore the tefillin loosely as shown in **Fig 1B**, respectively. Both groups were assisted in donning the tefillin. The raw data was then transmitted to the Kubios software as noted in **Fig 1C** and described below in detail.

### Data analysis using the Kubios software

All measurements were retrieved using Kubios HRV Premium software (Kubios Oy, Kuopio, Finland). The Kubios software derives the time domain measurements from the beat-to-beat RR intervals values. The frequency domain measurements are determined with the Fast Fourier Transformation (FFT) and the parametric autoregressive (AR) modeling [39]. The automatic beat correction application in the software was used to remove outliers. All measurements were analyzed in 5-minute windows according to the protocols set forth by the Task Force of the European Society of Cardiology the North American Society of Pacing Electrophysiology [38]. The windows used for statistical analysis were the last 5 minutes of each recording period.

### Measurements

#### Time domain measurements

Time domain measurements quantify the amount of HRV occurring during the monitoring period. SDNN is reported as the mean of the standard deviation of normal-to-normal (NN) beats of the designated time frame. The pNN50 measurement is reported as the percentage of adjacent NN intervals that differ from each other by more than 50ms. The RMSSD is measured as root mean square of successive RR interval differences while the HRV triangular index with is the integral of the density of the RR interval histogram divided by its height. The HRV triangular index is automatically calculated by the Kubios software, though technically it is inappropriate to use for short-term changes in HRV and should only be used in studies of 20 minutes or longer [38].

#### Frequency domain measurements

A Fast Fourier Transformation was used to convert the ECG units to hertz. The LF range includes all values between 0.04–0.15Hz while HF range includes all values between 0.15–0.40Hz, the power of each is defined by how much of the signal is located in that range.

#### Nonlinear domain measurements

Nonlinear Measurements were obtained by graphing a Poincaré plot. This graphs every R-R interval against the prior R-R interval creating a scatter plot. The R-R interval is the time between two adjacent R-waves on the ECG. An ellipse was created within the scatter plot. The long axis of the ellipse specifies SD2 measurements.

### Statistics

All statistical analysis was performed using SigmaStat (version 13, SPSS, Chicago, IL). Data are presented as means ± SEM for both absolute values and percent change relative to individual baseline. Any data points that were more than 2 standard deviations from the mean values were designated as outliers and removed from analysis. For multiple group comparison, we used repeat measures 2-way ANOVA with the Holm-Sidak method post hoc analysis where appropriate. Values of P ≤ 0.05 were considered statistically significant. All plots were created using Prism (GraphPad, San Diego, CA).

## Results

As shown in detail in **Table 1**, the average age of our study population was 23.9 years and was not different between the genders. Both control and tefillin groups consisted of 7 males and 8 females. The average ages were 25.43±5.50 (males) and 21.50±2.78 (females) for the control group and 24.86±3.29 (males) and 23.63±2.97 (females) for the tefillin group. Baseline values for all variables are presented by gender, there was greater weight and height in the males. Kubios software analysis of the H10 polar device recordings generated absolute values for each group and at the three time points (**Table 2)**. In addition, we compared the percent change from baseline to treatment (reactivity), and between treatment and post-treatment (recovery) as shown in **Table 3**.

**Table 1:** Demographics and baseline parameters for the subjects.

		Males	Females	All	P value (M vs F)
**Participants**		14	16	30	
**Age**		25.1±4.4	22.5±2.9	23.8±4.1	0.07
**Heart Rate (bpm)**		67.21±9.28	71.75±11.05	69.63±10.35	0.24
**Weight (lb)**		176.8±21.5	147.5±20.1	161.7±20.6	0.005*
**Height (in)**		70.0±3.7	65.7±2.7	67.8±3.3	0.0002*
**Time domain**	**SDNN (ms)**	65.8±32.5	58.3±32.6	61.8±32.2	0.53
	**pNN50 (%)**	30.5±25.8	31.4±22.6	31.0±23.7	0.92
	**RMSSD (ms)**	60.9±42.7	59.0±39.5	59.9±40.3	0.90
	**Triangular Index**	13.8±5.1	13.7±5.2	13.8±5.1	0.96
**Frequency domain**	**LF (nu)**	63.4±17.8	51.0±22.1	56.8±20.8	0.10
	**HF (nu)**	36.6±17.8	49.0±22.1	43.2±20.8	0.10
	**LF/HF (%)**	3.0±3.4	2.0±2.8	2.5±3.1	0.36
**Non-linear domain**	**SD1 (ms)**	43.1±30.3	41.8±28.0	42.4±28.6	0.90
	**SD2 (ms)**	81.2±37.8	70.3±38.3	75.4±37.9	0.44

Average values ±SEM.

*P<0.005 males compared to females. SDNN: Standard deviation of all R–R intervals; pNN50: Percentage of successive normal sinus RR intervals, RMSSD: Root mean square of successive differences, LF: Low frequencies, HF: High frequencies, LF/HF: Low frequencies/high-frequencies ratio, SD1: Standard deviation—Poincaré plot crosswise, SD2: Standard deviation—Poincaré plot lengthwise

**Table 2:** Average absolute values for HRV measurements.

		Control			Tefillin		
		Baseline	Treatment	Post-treatment	Baseline	Treatment	Post-treatment
**Time-domain**	**SDNN**	58.03±7.29	61.15±6.96	57.18±6.50	47.69±4.91	52.68±5.02	57.53±7.22*
	**pNN50**	31.055±5.88	38.30±6.55†	35.56±6.51	30.83±6.58	31.27±5.95	30.20±6.62
	**RMSSD**	49.87±6.64	60.96±8.88†	54.42±7.82	49.57±7.75	53.14±7.77	57.53±10.92
	**HRV Triangular index**	14.31±1.46	15.18±1.24	14.48±1.30	12.94±1.20	12.91±1.08	13.87±1.25
**Frequency-domain**	**LF**	59.65±4.84	51.09±5.89	53.42±5.82	56.44±5.80	61.58±3.40	66.22±4.58
	**HF**	40.34±4.83	48.89±5.88	46.57±5.82	43.53±5.80	38.39±3.4	33.76±4.58
	**LF/HF ratio**	1.88±0.42	1.098±0.24	1.25±0.31	1.23±0.28	1.58±0.27	2.17±0.43*
**Non-linear indices**	**SD1**	35.31±4.70	43.17±6.29	38.54±5.54	35.10±5.49	37.63±5.50	40.75±7.74
	**SD2**	73.48±9.61	73.92±8.39	70.01±8.12	58.49±6.07	65.99±5.81	71.70±8.19*

*P>0.05 for tefillin post-treatment compared to baseline. †P<0.05 for control treatment compared to control baseline.

Average values ±SEM. SDNN: Standard deviation of all R–R intervals; pNN50: Percentage of successive normal sinus RR intervals, RMSSD: Root mean square of successive differences, LF: Low frequencies, HF: High frequencies, LF/HF: Low frequencies/high-frequencies ratio, SD1: Standard deviation—Poincaré plot crosswise, SD2: Standard deviation—Poincaré plot lengthwise

**Table 3:** Average percent change from baseline for HRV measurements.

		Control		Tefillin	
		Reactivity	Recovery	Reactivity	Recovery
**Time-domain**	**SDNN**	1.30±5.22	5.14±7.56	9.43±3.37	19.34±7.07
	**pNN50**	12.82±10.57	-14.01±8.71	39.29±24.92	20.48±19.46**
	**RMSSD**	20.97±10.79	15.80±10.13	9.39±4.44	10.11±6.44
	**HRV Triangular index**	5.05±5.29	2.58±6.08	2.91±4.98	10.22±6.37
**Frequency-domain**	**LF**	-12.55±9.22	-5.41±11.07	11.29±6.33	28.74±11.98**§
	**HF**	22.72±14.10	14.42±13.86	-7.06±7.83	-23.13±6.4§
	**LF/HF ratio**	-19.40±21.15	-11.13±20.01	12.79±12.30	70.17±31.28**§
**Non-linear indices**	**SD1**	21.01±10.79	15.83±10.14	6.51±3.63	11.66±6.71
	**SD2**	-2.65±4.63	1.89±7.41	17.54±6.14#	20.65±7.45

#P<0.05 for control compared to tefillin during reactivity

**P<0.05 for reactivity compared to recovery for the tefillin group

§P<0.05 for control compared to tefillin during recovery

Average values ±SEM. SDNN: Standard deviation of all R–R intervals; pNN50: Percentage of successive normal sinus RR intervals, RMSSD: Root mean square of successive differences, LF: Low frequencies, HF: High frequencies, LF/HF: Low frequencies/high-frequencies ratio, SD1: Standard deviation–Poincaré plot crosswise, SD2: Standard deviation–Poincaré plot lengthwise

### Time domain (Fig 2)

**Fig 2:**
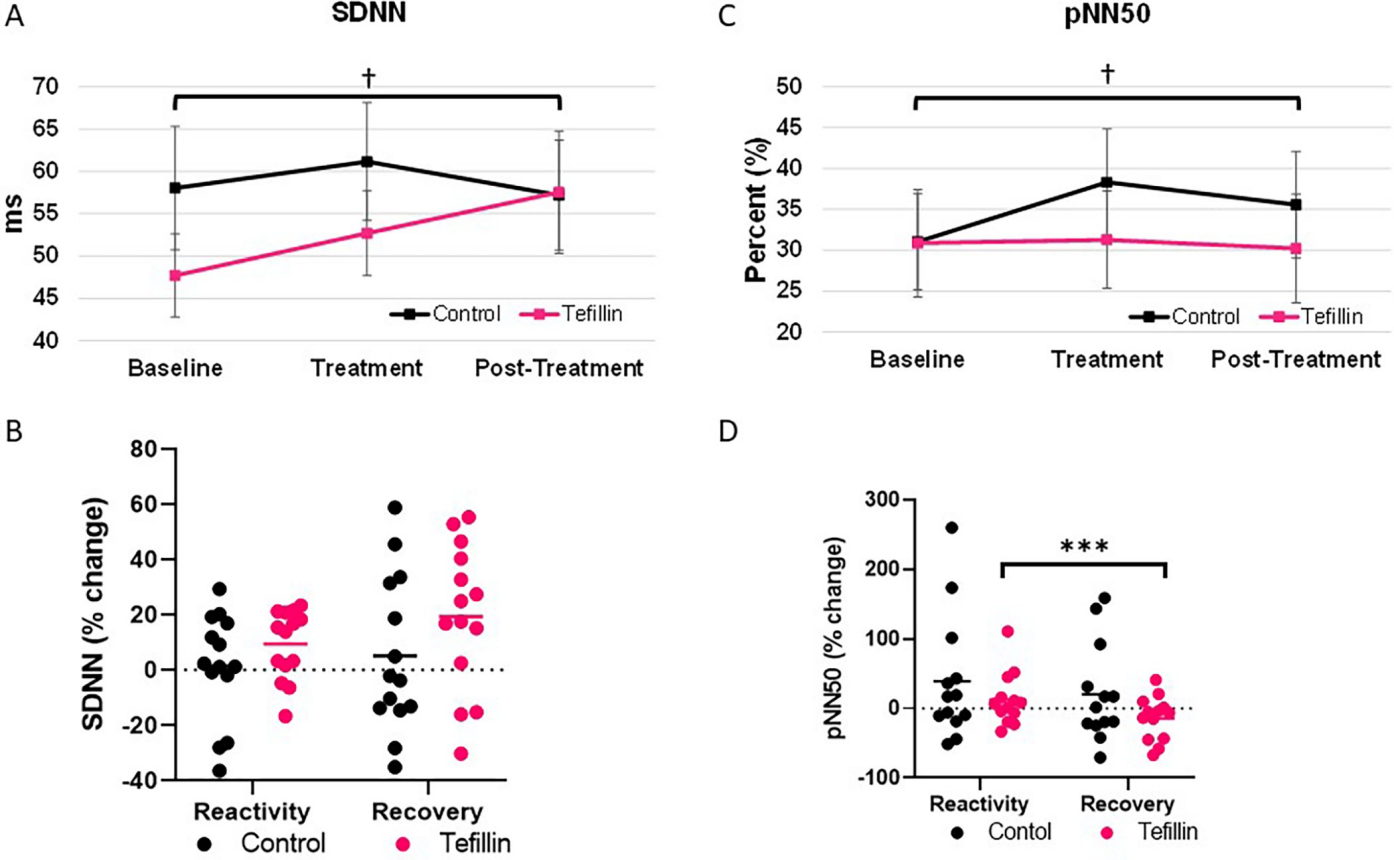
The Time domain measurements were determined for control and tefillin subjects. Standard deviation of all R-R intervals (SDNN) for **A**. absolute values and **B**. percent change. Percentage of successive normal sinus RR intervals (pNN50) for **C**. absolute values and **D**. percent change. Absolute data represents an average of subjects during baseline, treatment and post-treatment ±SEM. Reactivity and recovery are the average differences between the baseline and treatment as well as treatment and post-treatment ±SEM, respectively. †P<0.05 compared to baseline and ***P = 0.027 for tefillin reactivity compared to tefillin recovery.

Absolute values of SDNN increased in the tefillin group from baseline to post-treatment, 47.69±4.91 to 57.53±7.22 msec (P = 0.039), while no significant change was noted in the control group (P = 0.665) **(Fig 2A)**. There was no significant change in either group in relative terms in comparison to baseline, with a trend towards higher values in the recovery period in the tefillin (change of 17.6±6.8%; p = 0.103) **(Fig 2B)**. Furthermore, 11/15 subjects in the tefillin group had increased SDNN measured in recovery in comparison to baseline while only 7/15 did so in the control group. In contrast, absolute values of pNN50 increased in the control group from 31.06 ± 5.88 to 38.3 ± 6.55 percent during treatment (p = 0.024); while there were no significant changes noted in the tefillin group (p = 0.876) **(Fig 2C)**. However, the percent change in reactivity and recovery for the tefillin group decreased 24.94% (P = 0.027) with no significant difference between control values **(Fig 2D)**. No significant differences were observed in the RMSSD and the triangular index values for either group at any time point (**Tables 2 and 3**).

### Frequency domain (Fig 3)

**Fig 3:**
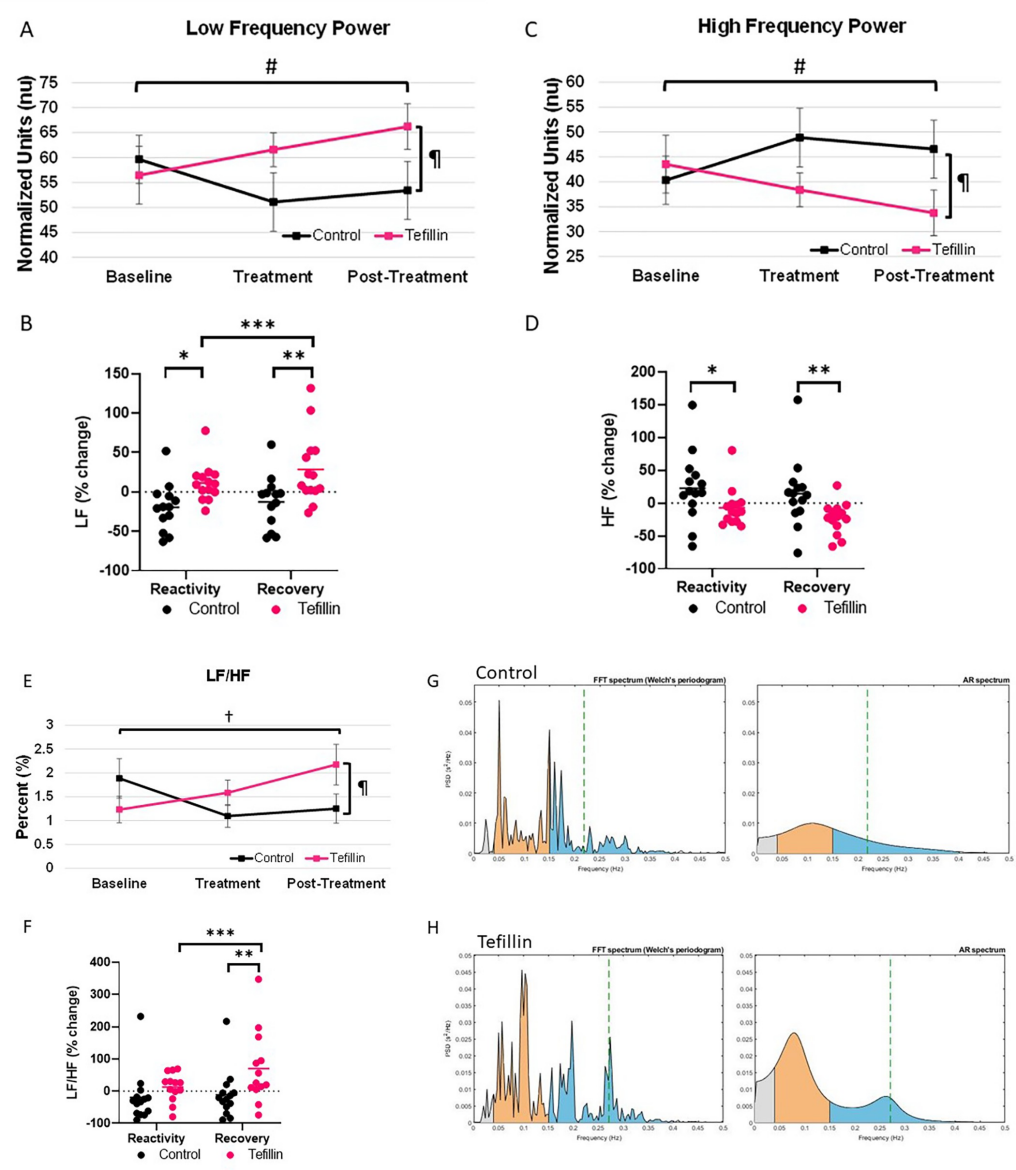
The frequency domain measurements were determined for control and tefillin subjects. Low frequencies (LF) for **A**. absolute values and **B**. percent change. High frequencies (HF) for **C**. absolute values and **D**. percent change. The ratio of LF/HF for **E**. absolute values and **F**. percent change. Absolute data represents an average of subjects during baseline, treatment and post-treatment ±SEM. Reactivity and recovery are the average differences between the baseline and treatment and treatment and post-treatment ±SEM, respectively. #P = 0.097 for tefillin baseline compared to tefillin post-treatment; ¶P = 0.087 for control compared to tefillin at post-treatment; *P<0.05 for control versus tefillin at reactivity; **P<0.05 for control versus tefillin at recovery; and ***P<0.05 for tefillin reactivity compared to tefillin recovery. Representative graphs from the Kubios software for **G**. control and **H**. tefillin at post-treatment, with LF peaks in orange and HF peaks in blue.

The determination of LF and HF power was performed by converting the ECG units to Hertz and then to normalized units by the Kubios software, as described in the methods. The two main frequency dependent variables, LF and HF, demonstrated changes during treatment in the tefillin group. Specifically, LF increased in the tefillin group from 56.44±5.80 to 61.58±3.4 nu (p = 0.097) during treatment and was higher than control during post-treatment (LF 56.44±5.80 vs 66.22±4.58 nu p = 0.087) **(Fig 3A)**. In relative terms, the changes were more apparent with tefillin increasing LF in comparison to control at both reactivity (p = 0.025) and recovery (p = 0.004) periods as well as within reactivity to recovery (p = 0.01) (**Fig 3B**). Further, LF increased in 13/15 subjects in the tefillin group and 5/15 in the control group during reactivity.

In contrast, the HF power decreased in the tefillin from 43.53±5.8 nu at baseline to 38.39 ± 3.4 nu during treatment (p = 0.097) and to 33.76±4.58 (p = 0.087) in post-treatment **(Fig 3C).** In relative terms, the control group increased by 22% while the tefillin group dropped by 7% during reactivity (P = 0.049) and the control increased 14% during recovery in comparison to the decrease of 23% in the tefillin group (P = 0.014) (**Fig 3D**).

The ratio of LF/HF power decreased in the control group during and post-treatment (p = 0.055 for each), while it increased dramatically in the tefillin group during treatment and post-treatment from 1.23±0.28 at baseline to 1.58±0.27 and 2.17±0.43 (P = 0.016 for post vs. baseline) **(Fig 3E)**. This finding was also found in relative terms with tefillin increasing 13% during reactivity (p = 0.309 vs control) and 70% in recovery (P = 0.013 vs control) **(Fig 3F).** Furthermore, the ratio increased in nearly all subjects in the tefillin group (13/15) with only 5/15 increasing in the control group. Representative graphs are noted in **Fig 3G** for a subject at baseline and **Fig 3H** after tefillin treatment.

### Nonlinear markers (Fig 4)

**Fig 4:**
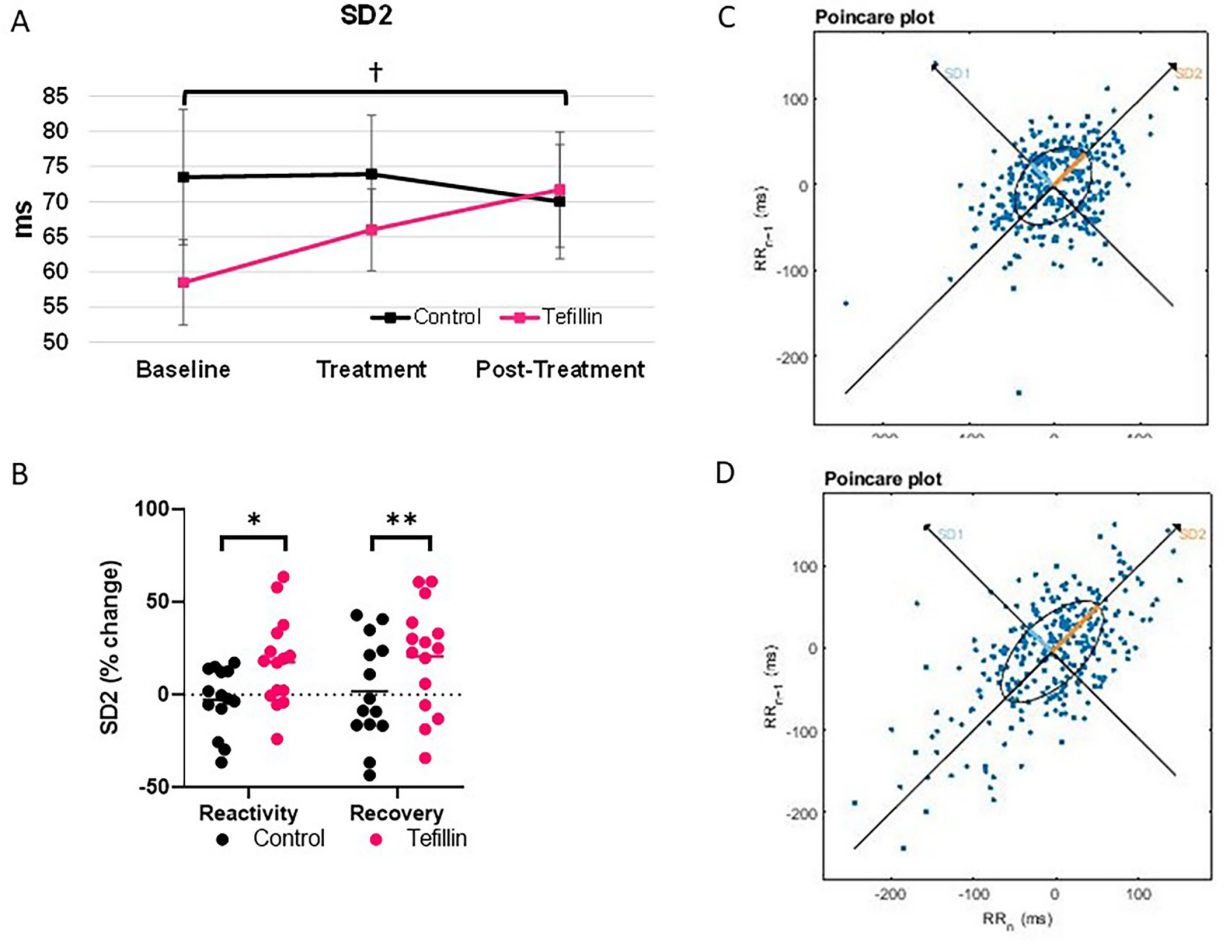
The frequency domain measurements were determined for control and tefillin subjects. Standard deviation–Poincaré plot lengthwise (SD2) for A. absolute values and B. percent change. Absolute data represents an average of subjects during baseline, treatment and post-treatment ±SEM. Reactivity and recovery are the average differences between the baseline and treatment and treatment and post-treatment ±SEM, respectively. †P = 0.041 for tefillin baseline compared to tefillin post-treatment; *P<0.05 for control versus tefillin at reactivity; and **P<0.05 for control versus tefillin at recovery. Representative Poincaré plots from the Kubios software for C. tefillin at baseline and D. tefillin at post-treatment.

No significant differences were noted in either group at any time point as it pertained to SD1 **(Tables 2 and 3)**. In contrast, the tefillin group saw increased SD2 values in comparison to baseline on an absolute and relative basis. Specifically, there was an increase from 58.49±6.07 to 65.99±5.81 to 71.70±8.19 (**Fig 4A)** which represented a change of 18% and 21% in the tefillin group while the control group exhibited virtually no change in this parameter at any time point (maximal change -2.7% during reactivity and 1.9% during recovery) (P = 0.034 and P = 0.048 in comparison to tefillin, respectively) (**Fig 4B**). In summary, 11/15 subjects in the tefillin group and only 7/15 subjects in the control group demonstrated an improvement in SD2 values. Representative images of the Poincare plots before and after tefillin are shown in **Fig 4C** and **4D**.

## Discussion

NOB via tefillin use quickly induces changes in some HRV derived parameters in healthy males and females in comparison to baseline as well as control, as evidenced by increases in SDNN, LF, LF/HF and SD2 and a decrease in HF.

Previous work in the RIPC/HRV field have yielded conflicting results, partially due to differences in RIPC techniques and study populations. For example, Telles et al. [25] found that “high pressure” RIPC resulted in increased LF, decreased HF and an increase in the LF/HF ratio. Morley et al. [26] found a similar pattern when exercise was added to RIPC as did Zagidullin et al. with traditional RIPC [27], though the latter did not reach statistical significance. Interestingly, not all data regarding LF and HF parameters followed this pattern. Others, such as Gardner et al. [28] reported that up to 12 sessions of 40 minutes each of cycles of ischemia/reperfusion via blood pressure (BP) cuff inflation/deflation were required to exert statistically significant *decreases* in LF as well as the LF/HF ratio (while a single use produced a smaller, but similar effect) and Khaliulin et al. [29] showed that RIPC *blunted* the rise of LF during stressful conditions. However, in our study (and similarly to [25–27]) the most striking change was an *increase* in these values in the tefillin treated group with a decrease in the control group after a single intervention [28].

Variations on the technique and population have also been reported. For example, Sabino-Carvalho et al. [30] performed similar RIPC stimulation (though on the thigh) and found minimal changes in HRV parameters after a single intervention in endurance runners, and Zagidullin and colleagues as reported above [27] also found that RIPC stimulation on the forearm had no demonstrable effects on HRV in healthy subjects, though some changes were observed in subjects with prior coronary artery disease. Interestingly, Chen and colleagues [31] studied subjects with “mild and stable” heart failure and found that a 6-week course of RIPC through BP cuff inflation/deflation on the upper arm improved clinical and virtually all HRV dependent markers though LF was decreased, HF was increased and the ratio therefore decreased, potentially due to alterations in PNS tone is subjects with heart failure [31]. In contrast, Cho and colleagues [32] found LF increased in healthy volunteers, but not in subjects undergoing cardiac surgery (HF was not reported). In summary, variability with regards to methods of induction of RIPC as well as target population have resulted in contradictory data as it pertains to changes in LF and HF variables. That being stated, there appears to be agreement that there are measurable changes under most of these conditions that may have important clinical implications.

Time domain markers of HRV such as SDNN have been shown to reflect both sympathetic and parasympathetic tone while RMSSD and pNN50 are measures of parasympathetic activity [40]. In this study, we found that SDNN increased in the treatment group, but no change was observed in the control group. On the other hand, pNN50 increased with control subjects, while no changes were observed in RMSSD. As described above, most prior studies did not find a change in these parameters after a single intervention, though those that reported changes after many interventions [6] also described that SDNN increased in their treatment groups. This was similar to what was observed in our study and consistent with the concept that tefillin use affected both PNS and SNS while control increased solely PNS tone consistent with increased relaxation.

In contrast to the studies that have observed a change in linear values, our study found an increase in LF and a decrease in HF in the tefillin group with the opposite effect observed in the control group. Thus, These effects could potentially be interpreted as harmful, though in one of the few animal studies where HRV dependent variables were collected prior to infarct, Abdul-Ghani and colleagues [41] found that 30 minutes following an RIPC stimulus there was an increase in LF/HF ratio in the preconditioned mice that subsequently had decreased infarct size. This finding was similar to that reported by Bonaduce and colleagues [42] in patients with chronic heart failure that found that those with higher LF/HF ratio had dramatically improved survival in comparison to those with a lower ratio.

These findings are further supported by the non-linear measures where we found no changes in either group in SD1, a marker of short term HRV and baroreceptor activity. However, the tefillin treated group had an increase in SD2 consistent with long term HRV and associated (via SD1 to SD2 ratio) with improved outcomes in patients with evolving acute coronary events [43] and those with heart failure [42].

The data shown in this study is consistent with our prior report on the use of tefillin where we also demonstrated improvement in surrogate markers of RIPC after single use and within a very short time (as opposed to many other studies that have measured HRV dependent variables days and in some cases weeks after the stimulus) [27–30]. Thus, in summary it appears that tefillin use affects both PNS and SNS tone, though the duration of the effect and its likely compounding effects on markers such as SDNN, SD2 and LF will need to be parsed out further in healthy subjects as well as those with cardiovascular disease.

The increasing data in this field has significant clinical implications as the field of RIPC has seen a number of high quality, international studies demonstrate no significant benefit for its use in subjects with ischemic heart disease [15, 29]. These studies focused on repeated bouts of cuff deflation and inflation in subjects with prior and ongoing coronary artery disease, while our studies (and others described above) differ in two key aspects that can potentially explain the discrepancy between the decades strong animal data demonstrating a very strong cardioprotective effect and the lack of protection in human subjects.

First, the animal studies and (most preclinical studies including ours) have applied the preconditioning stimulus prior to an ischemic event, while almost all clinical studies applied the stimulus during (or after) the ischemic event. This aspect appears even more critical in light of our data demonstrating that a RIPC phenotype can be elicited quickly and efficiently prior to an ischemic event, but its use during an event is questionable. Further, our observation that the LF/HF ratio and SD2 are increased only in the tefillin group argues that attempting to elicit the effect during a coronary event is unlikely to provide further benefit to the subject (as the event is actively unfolding and causing these changes as the heart damage is ongoing).

Second, most of studies have employed an inflation/deflation system with inflations of up to 20 mmHg above systolic [15, 29]. The majority of the preclinical studies showed that the effect needed to be repeated several times before a measurable change was detected [28], thus, strongly arguing for the difficulty in applying this method of RIPC in a clinically relevant matter and contrasting with our observation that tefillin use induces these changes promptly and with a single use.

While this study did not directly address the mechanistic basis of this effect, it does demonstrate that the sensation of pressure on the skin is at least partially associated with the observed effects. This is consistent with our animal data that demonstrated that NOB is sufficient to elicit a cardioprotective response, while blockade of the sensation blunted the observed cardioprotection [20]. This finding is further in line with data from Jones et al. that documented that capsaicin stimulation of TRPV1 channels was sufficient to elicit a cardioprotective response [12]. Thus, it appears that the skin/pressure/pain connection that was previously described by Caterina and Julius [44] extends beyond to include parasympathetic and sympathetic modulation of cardiac function.

Finally, these effects add further supporting data that tefillin use itself, likely through NOB induced RIPC changes in inflammatory, vascular and parasympathetic tone, was a key determining factor in the population-based studies that found improved cardiovascular outcomes in orthodox Jewish males as opposed to both orthodox females and non-orthodox Jews that do not traditionally wear tefillin on a nearly daily basis [25, 45]. In sum, it appears that the observed RIPC effect in humans has overlapping anti-inflammatory effects (as we have previously described) together with changes in PNS and SNS tone that this study further elucidates as described above in detail.

Future studies will attempt to tease apart the overlapping roles of wearing tefillin with other confounding factors such as resting (which improved parasympathetic tone in our control group), meditation (which has also been shown to improve HRV dependent markers) and prayer (which may in and of itself affect physiologic variables). Future studies will also evaluate the effect of chronic tefillin use both in healthy populations as well as those with prior ischemic conditions.

### Limitations

This clinical study was designed to use a HRV monitoring device on healthy human volunteers in order to determine if there was a correlation between our previous studies using tefillin [20, 23] and changes in HRV parameters. Though we attempted to eliminate many stimuli that can affect HRV (exclusion criteria listed in Study Design), there are additional factors found in the literature that were not accounted for in this small preliminary study [33, 34, 46, 47]. Further, while we attempted to replicate the exact wearing of tefillin on all subjects there is likely to be minor variability in the degree of pressure applied to each subject.

## Conclusions

Non-obstructive banding via tefillin use affects HRV dependent variables that have been associated with improved outcomes both before and after ischemic events. This finding provides further evidence that RIPC through NOB may still be a viable clinical pathway to prevent and decrease the morbidity associated with ischemic events.
